# Carbon storage in soils of Southeastern Nigeria under different management practices

**DOI:** 10.1186/1750-0680-5-5

**Published:** 2010-09-26

**Authors:** Martin AN Anikwe

**Affiliations:** 1 Department of Agronomy and Ecological Management, Faculty of Agriculture and Natural Resources Management, Enugu State University of Science and Technology, P.M.B. 01660 Enugu, Nigeria

## Abstract

**Background:**

Changes in agricultural practices-notably changes in crop varieties, application of fertilizer and manure, rotation and tillage practices-influence how much and at what rate carbon is stored in, or released from, soils. Quantification of the impacts of land use on carbon stocks in sub-Saharan Africa is challenging because of the spatial heterogeneity of soil, climate, management conditions, and due to the lack of data on soil carbon pools of most common agroecosystems. This paper provides data on soil carbon stocks that were collected at 10 sites in southeastern Nigeria to characterize the impact of soil management practices.

**Results:**

The highest carbon stocks, 7906-9510 gC m^-2^, were found at the sites representing natural forest, artificial forest and artificial grassland ecosystems. Continuously cropped and conventionally tilled soils had about 70% lower carbon stock (1978-2822 gC m^-2^). Thus, the soil carbon stock in a 45-year old *Gmelina *forest was 8987 gC m^-2^, whereas the parts of this forest, that were cleared and continuously cultivated for 15 years, had 75% lower carbon stock (1978 gC m^-2^). The carbon stock of continuously cropped and conventionally tilled soils was also 25% lower than the carbon stock of the soil cultivated by use of conservation tillage.

**Conclusion:**

Introducing conservation tillage practices may reduce the loss of soil carbon stocks associated with land conversion. However, the positive effect of conservation tillage is not comparable to the negative effect of land conversion, and may not result in significant accumulation of carbon in southeastern Nigeria soils.

## Background

Soil organic carbon is a large and active pool, containing roughly twice as much carbon as the atmosphere and 2.5 times as much as the biota. Carbon sequestration is the facilitated redistribution of carbon from the air to other pools. This would reduce the rate of atmospheric CO_2 _increase, thereby mitigating global warming [1,2].

The amount of carbon sequestered at a site reflects the long-term balance between influx and efflux of carbon. Recent concerns with rising atmospheric levels of CO_2 _have stimulated interest in C flow in terrestrial ecosystems and the latter's potential for increased soil carbon sequestration [3]. Carbon enters the soil as roots, litter, harvest residues, and animal manure. It is stored primarily as soil organic matter (SOM). The density (w/v) of carbon is highest near the surface, but SOM decomposes rapidly, releasing CO_2 _to the atmosphere. Some carbon becomes stabilized, especially in the lower part of the profile. However, in many areas, agricultural and other land use activities have upset the natural balance in the soil carbon cycle, contributing to an alarming increase in carbon release [4,5]. Since the current rise in atmospheric CO_2 _is thought to be mitigated in part by carbon sequestration in agricultural soils [4], interest has increased in the possible impacts of various agricultural management practices on soil organic matter dynamics [6].

Agricultural and other land use practices have a significant influence on how much carbon can be sequestered and how long it can be stored in the soil before it is returned to the atmosphere. The best strategies focus on the protection of soil organic carbon against further depletion and erosion, or the replenishment of depleted carbon stocks through certain management techniques [2]. In either case, the keys to successful soil carbon sequestration are increased plant growth and productivity, increased net primary production and decreased decomposition [2]. Similarly, conversion of marginal arable land to forestry or grassland can rapidly increase soil carbon sequestration. For example, analysis of long-term crop experiments indicated that increasing crop rotation complexity increased SOC sequestration by 20 gC m^-2 ^yr^-1^, on average [7]. In long-term experiments in Canada, SOC sequestration rates were 50 to 75 gC m^-2 ^yr^-1 ^in well-fertilized soils with optimal cropping [8]. By contrast, long-term experiments in the northern Great Plains (US) have shown that fertilizer N increased crop residue returns to the soil, but generally did not increase SOC sequestration [9]. Ogunwale and Raji [8] found that after 45 years of cow dung and NPK treatments to a soil in Samaru Northern Nigeria, soil organic carbon content in the unamended soil was 1.81 tC ha^-1 ^or 10 gC m^-2 ^between 1977 and 1995. In the same period of 45 years, the use of continuous NPK application resulted in only slight increase in SOC (3%) over the unamended soil while manure with NPK gave 115% more SOC. They found that the rate of SOC sequestration during fallow period in their experiment was approximately 400% more than the rates under continuous cultivation.

Timing and intensity of tillage also must be taken into account in the design of best management practices for maximizing SOC sequestration [10-12].

In most of Africa including Nigeria, research on quantification of carbon stored in the soil is proceeding slowly. Thus, data on soil C pools are lacking for most common agro-ecosystems. It is important to note that data collected from tropical environments are used in estimating total world carbon sequestration potential because differences in edaphoclimatic conditions and soil management practices influence the storage of carbon in the soil. For example, with the exception of histosols that have 13-27% soil organic matter (by weight) [13], average soil organic matter contents of soils in sub-Saharan Africa range (between) 0.5-3.0% whereas temperate Europe and America soils record up to 10-13% soil organic matter. Quantifying changes in soil C is a difficult task. Annual changes per year are small compared to C already present, and its spatial variability can be very large [14]. Thus, reliable estimates of C change depend on sampling randomly at test sites over many years or by sampling at specific locations, repeatedly over time [15].

African countries are unlikely to engage in soil carbon sequestration unless there are clear local economic and societal benefits. Therefore, it is essential to estimate all potential costs and benefits related to the various management options. Large-scale adoptions of ecologically sound land use practices are likely to be the most cost effective and environmentally friendly option to increase soil carbon sequestration in Africa [2]. In addition, a correct measurement and verification of carbon sequestration potential of soils in sub-Saharan Africa would enable the zone to participate in the Clean Development Mechanism (CDM), proposed in Article 12 of the Kyoto Protocol to the United Nations Framework Convention on Climate Change. This will allow developing countries to sell or trade project-based carbon credits, such as Carbon Emission Reduction (CER) credits, to or with industrial countries, if adopted. CER credits could provide an incentive for participation in climate change mitigation and cover the costs that African participants will encounter when engaging in carbon sequestration [2].

The objective of this work, therefore, is to assess quantitatively, the effect of different soil management practices on soil organic carbon sequestration.

## Results

### Soil properties of the study sites

Results of the study (Table 1) indicate low, medium and high coefficients of variability among soil properties at the different sites studied. There was a low coefficient of variability (6-9%) in bulk density and soil pH in CaCl_2 _at the different soil depths studied, whereas silt + clay content and percent sand content showed medium variability (20-30%). The highest SOC content (3.07%) was found in site No. 6 (natural undisturbed forest) (Table 2), whereas lowest S0C was observed in site No. 10 (conventionally-tilled, continuously-cropped plot (CT-CC) (0.81%) S0C and site No. 2 (CT-CC Plot) (0.83%) (SOC). Lowest SOC levels were found in sites 2, 5 and 10 (CT-CC plots) with SOC range of between 0.59-0.83%. Ratings by Landon [16] in the study area show 1.16% SOC or lower to be low, whereas SOC values ≥ 1.74% and above are regarded as high. Sites 2 and 10 as shown in Table 1, were conventionally-tilled and continuously cropped soils.

**Table 1:** Selected Soil Properties of the Study Sites

S/N	Bulk	Density	**(Mgm**^**-3**^**)**	Organic	Carbon	**(MgKg**^**-1**^**)**	Total	N	**(MgKg**^**-1**^**)**	pH	**(Cacl**_**2**_**)**		Clay + silt	**MgKg**^**-1**^		Sand	**MgKg**^**-1**^	
	0-5	5-15	15-30	0-5	5-15	15-30	0-5	5-15	15-30	0-5	5-15	15-30	0-5	5-15	15-30	0-5	5-15	15-30
1.	1.47	1.47	1.49	1.25	1.22	1.20	0.126	0.111	0.101	5.0	5.0	5.3	56	56	58	44	44	42
2.	1.52	1.55	1.56	0.83	0.80	0.61	0.070	0.041	0.036	4.9	5.2	5.2	36	34	34	64	66	66
3.	1.30	1.32	1.37	2.31	2.13	1.98	0.130	0.126	0.120	5.3	5.3	5.5	26	28	28	74	72	72
4.	1.36	1.37	1.39	2.79	2.70	2.44	0.24	0.211	0.18	4.8	4.6	4.6	38	38	40	62	62	60
5.	1.29	1.31	1.32	0.63	0.62	0.59	0.09	0.09	0.07	4.3	4.3	4.6	34	35	38	66	65	62
6.	1.31	1.32	1.35	3.07	2.90	2.72	1.95	1.90	1.61	4.5	4.7	4.7	36	37	37	64	63	63
7.	1.23	1.25	1.28	2.32	2.46	2.66	0.20	0.20	0.15	4.9	4.6	4.5	37	37	39	63	63	61
8.	1.60	1.62	1.66	1.63	1.57	1.52	0.27	0.22	0.21	5.2	5.4	5.4	28	30	30	72	70	70
9.	1.44	1.45	1.48	1.06	1.00	0.94	0.085	0.082	0.080	5.04	5.0	4.5	35	37	37	65	63	63
10.	1.49	1.51	1.54	0.81	0.72	0.70	0.042	0.040	0.040	4.4	4.5	4.5	66	68	69	34	32	31
																		

CV (%)	8.6	8.5	8.39	53.3	54.1	55.7	178	186	184	6.9	7.67	8.00	31.5	30.9	42.00	29.3	20.6	21.5

**Table 2:** Total quantity of soil organic carbon (gC m^-2^) stored at the 0-30 cm soil layer of the study soils

Site Number	0-5 (Mean + SEM)	Soil Depth 5-15 (Mean + SEM)	15-30 (Mean + SEM)	Total
1.	918.8 ± 12	1793.4 ± 2	1788 ± 44	4500.2
2.	630.8 ± 28	1240 ± 15	951.6 ± 23	2822.4
3.	1501.5 ± 34	2811.6 ± 33	2712.6 ± 20	7025.7
4.	1897.2 ± 26	3699 ± 18	3391.6 ± 26	8987.8
5.	387.5 ± 18	812.2 ± 15	778.8 ± 31	1978.5
6.	2010.9 ± 15	3828 ± 30	3672 ± 15	9510.9
7.	1426.8 ± 24	3075 ± 42	3404.8 ± 56	7906.6
8.	1308 ± 33	2551.5 ± 46	2523.2 ± 13	6382.7
9.	763.2 ± 12	1450 ± 21	1391.2 ± 17	3604.4
10.	603.5 ± 30	1087.2 ± 34	1078 ± 20	2768.7

CV (%)	65.3	54.5	56.3	55.1%

The highest total N content of the soils ranged from 0.29-1.95 Mg kg^-1^. These were found at sites 8, 7 and 6. These plots were either artificially planted forests or natural undisturbed forests (Table 3), whereas sites 2, 10 and 5 had low N content, and correspond to plots that were conventionally- and continuously-tilled. Results show slight differences in pH values for the different soils studied. However, sites 5 and 10, which were continuously- and conventionally-tilled plots, were among the plots with the lowest soil pH.

**Table 3:** Location, classification and land use history of the 10 sites used for the study

Site Number	Location/Annual rainfall	Soil Classification	Land use history
1	Abakaliki I 6°19'N,8°06'E 2069 mm	Aquept Flood Plain	Conventionally-tilled with traditional hoes, planted with Cassava {*Manihot esculenta}*/vegetables (Amaranth {*Amaranthus hybridus}*, Okra {*Albemoschus esculentus}*,Waterleaf {*Talinum triangulare} *)/maize {*Zea mays*} intercrop with 2-year fallow period in 10 years, no fertilization, crop residues not removed.

2	Enugu I 6°27'N,7°29'E 1792 mm	Typic Paleustult Midslope	Conventionally-tilled with traditional hoes, continuously-cropped with maize/cassava/yam {*Dioscorea rotundata*} intercrop for ten years. NPK 15:15:15 fertilizers used at low doses (30-50 kg ha^-1^), crop residues left in the field.

3	PortHarcourt 4°46'N,7°01E 2450 mm	Typic Paleustult Floodplain	Conventionally-tilled, unmulched, cropped to maize and cassava for 12 consecutive years, fertilized with low dose (30-50 kg ha^-1^) of NPK 15:15:15 fertilizer, crop residues left in the field.

4	Enugu II 06° 27'N,7°32'E 1792 mm	Typic paleustult Midslope	Artificial forest established by Forestry Department in 1962. Planted with *Gmelina arborea *and *Tectona grandis *(Teak).

5	Enugu III 06° 27'N,7°32'E 1792 mm	Typic paleustult Midslope	Adjacent land near the artificial forest cropped-continuously for 15 years with cassava, yam, pulses and vegetables in a mixed culture. No fertilization and crop residues not removed.

6	Ihe,Awgu I 06° 30'N,7°15'E 1752 mm	Typic paleudult Toeslope	Natural undisturbed forest (sacred land). Had existed for more than 80 years. People are forbidden entry. Hunting of animals/games and cutting of trees/fetching of firewood not allowed.

7	Enugu IV 06° 27'N,7°25'E 1750 mm	Typic Paleudult Midslope	Artificial grassland (golf course) established in 1934. Mainly made up of *Paspalum notatum*, *Axonopus compressus* and *Cyperus rotundus*. Regularly cut and fertilized with N:P:K 15:15:15 fertilizer.

8	Abakaliki II 6°04'N,8°65'E 2069 mm	Typic Haplaudult Crest	Artificial *Gmelina arborea *forest established 30 years ago. The alleys between the trees are currently cropped with different food crops(cocoyam, yam, cowpea, maize) by urban farmers. Municipal wastes used for fertilization, conventionally-tilled.

9	Nsukka, I 6°52'N,7°24'E 1700 mm	Typic Paleustult Footslope	University Research plot, fallowed for two years, conservation tillage, planted with maize and groundnut, fertilization with NPK 15:15:15 at 90 kg ha^-1 ^and poultry droppings at 10 Mg ha^-1 ^for three years.

10	Ihe,Awgu II 06° 30'N,7°05'E 1752 mm	Typic Paleudult Toeslope	Farmers plot, conventionally-tilled, planted with cassava/maize/vegetables (fluted pumpkins, cabbage, cowpea, Amaranth) with 2-year fallow interval, NPK 15:15:15 fertilizers used at low doses (30-50 kg ha^-1^), crop residues left in the field, farm managed as stated for 12 years.

### Quantity of carbon stock in the soils under different soil management regimes

Results of the study show that there were differences in total quantity of carbon sequestered in the different land utilization types in the study area (Table 2). These differences were confirmed by the high coefficient of variation (55%) between the SOC content of the different land use types.

The highest quantities of SOC were stored in sites 6, 4 and 7 with 9510.9, 8987.8 and 7906.6 gC m^-2 ^in the 0-30 cm soil layers, respectively (Table 2). These sites correspond to natural undisturbed forest, artificial forest and artificial grassland, respectively. Only slight differences in carbon stock (absolute difference between maximum and minimum value:1604 gC m^-2^) were found between the three land uses with the highest carbon stocks and that may be either because of differences in plant biodiversity, differences in bulk densities of the soils studied or slight differences in local climatic regimes.

The lowest carbon stocks in the 10 study locations were found in sites 5, 2 and 10. These have SOC stocks of 1978.5, 2822.4 and 2768.7 gC m^-2 ^in their 0-30 cm soil layer, respectively. These plots correspond to conventionally-tilled and continuously-cropped plots. When compared to the sites with the highest carbon stocks (forest and grassland land use types), results show 71% depletion in carbon stock in the conventionally-tilled, and continuously-cropped plots. More specifically, the quantity of carbon sequestered in site 4 (planted forest) was 8989.8 gC m^-2^. This was higher than that stored in an adjacent cultivated site (site 5) by as much as 78% (Table 2). Assuming that this forest reached a steady-state condition (balanced input and output of SOC), it took 15 years of continuous cultivation and conventional tillage to lose 78% of its carbon stock built over the years.

Results show that at site 8 (Abakaliki, Artificial *Gmelina arborea *forest with alleys cultivated with food crops), the quantity of carbon sequestered was 6382.7 gC m^-2 ^at 0-30 cm soil depth. This quantity was higher than the carbon stock found in site 1 (another Abakaliki plot, conventionally-tilled and continuously-cropped by 30%). In contrast, only a slight difference (5%) in total carbon stock was found between site 6 (natural undisturbed forest and site 4 (artificial forest).

## Discussion

High coefficients of variability in organic carbon and total N content were observed for soil organic carbon (SOC; 53-55%) and between 178-184% for total N. High variability in SOC and total N content may indicate soil properties that are mostly impacted on the short to medium term by changes in soil management practices. Although measured values of bulk density even among the same soil vary considerably because densification of surface soil is caused by many factors viz. trafficking by humans and animals, wetting and drying cycles in soils, raindrop impact energy, etc. [17], the low coefficient of variation observed among the different soils used for the study especially in cultivated plots, may come from the fact that samples were collected at the end of the harvesting season when soil re-compaction after tillage may have occurred. However, bulk density values are most useful in carbon sequestration studies for the calculation of total quantities of carbon sequestered at a particular time and soil depth. Krull et al. [18] stated that almost all organic carbon in soil is located within pores between mineral particles either as discrete particles or as molecules adsorbed onto the surfaces of these mineral particles. Soil architecture can influence biological stability of organic materials through its effects on water and oxygen availability, entrapment and isolation from decomposers, and through the dynamics of soil aggregation.

The highest SOC content was found in natural undisturbed forest, whereas lowest S0C was observed in conventionally-tilled, continuously-cropped plots. Previous studies by [17] and [19] showed that tillage adversely affects carbon storage in the soil. However, although sites 3 and 9 were continuously-tilled plots, their SOC contents were considerably high (2.3 and 1.06% in the 0-5 cm soil layer, respectively) when compared to sites either under grassland or forests probably because site 3 is a natural floodplain (see Table 3) whereby it seemed that enrichment of SOC occurred during yearly flooding. For site 9 in particular, the plot was managed under conservation tillage with annual addition of 20 t ha^-1 ^of poultry droppings for 3 years. These may have drastically increased SOC of sites 3 and 9. Differences in SOC content of site 4 (Artificial *Gmelina arborea *forest) and site 5 (adjacent CT-CC plot) show that land clearing and continuous cultivation drastically reduce SOC. Bationo et al. [20] in studying soil organic carbon dynamics, functions and management in West African agro-ecosystems reported rapid decline of SOC levels with continuous cultivation. For the sandy soils, they found that average annual losses may be as high as 4.7% whereas with sandy loam soils, losses were lower, with an average of 2%. They postulated that total system carbon in different vegetation and land use types indicated that forests, woodland and parkland had the highest total and aboveground carbon content demonstrating potential for carbon sequestration. For example, total system carbon in the Senegal River valley was 115 ton ha^-1^ in the forest zone and only 18 ton ha^-1 ^when the land was under cultivation. Cultivated systems have reduced carbon contents due to reduced tree cover and increased mineralization due to surface disturbance.

Generally, it seemed that SOC reduced with sampling depth at all sites used for the study. The continuously- and conventionally-tilled plots were among the plots with the lowest soil pH probably because of mining of exchangeable cations by growing crops in continuously-tilled plots. Generally, soil pH increased with soil depth in most of the sites studied. Mineralogy, surface charge characteristics, and precipitation of amorphous Fe and Al oxides on clay mineral surfaces define the capacity of clay minerals to adsorb and potentially protect SOC [21].

Results of this study also indicate that although site 3 was conventionally-tilled and cultivated for 12 consecutive years, it stored up to 7025 gC m^-2^. This may be because crop residues were always left in the field after harvesting but more importantly because it is a floodplain. It is likely that soil materials including C may have been transported from other places and deposited there. However, for site 9 (fallowed for 2 years, conservation till + fertilizer + poultry droppings and planted with maize) carbon stock was 3604 gC m^-2^, which was higher than the C values for plots 10 and 2 (conventionally-tilled, continuously-cropped plots) by up to 23%.

The quantity of carbon stored in the natural forest was greater than that of the artificial forest by 5% probably because of greater diversity of plant species found at the natural forests and to a lesser extent because the natural forests are older than the artificial forests. However, [21] and [22] have shown that both natural and artificial forest attain steady-state conditions after several years and thereafter only slight changes in SOC content are possible unless extraneous factors like climatic shifts occur.

These results show that conventional tillage reduces soil carbon stocks when compared to other management practices. However, the amounts and rates of carbon sequestration vary according to natural factors such as climate (temperature and rainfall) and soil physical characteristics (soil texture, clay mineralogy and soil depth) as well as agricultural management practices.

## Conclusion

The results of this study have shown that different management systems impact on the ability of the soil to sequester carbon. In tropical hot climates as those found in the study area, natural undisturbed forests, artificial forests and grasslands store between 7906-9510 gC m^-2 ^within the first 0-30 cm soil layer, whereas cultivated and continuously-cropped lands sequester about 1978-3604 gC m^-2 ^depending on the management system adopted. In other words, the large-scale conversion of forests to croplands in the southeastern Nigeria may lead to 50-75% loss in the regional soil carbon stock.

## Methods

### Site description

Soil samples were collected from 10 sites in different parts of southeastern Nigeria. Differences in management practices and edaphoclimatic properties guided choice of the different sites. Southeastern Nigeria stretches from 04°15'N to 07°00'N and between 05°34'E and 09°24'E, has a total area of approximately 78,612 km^2 ^[23]. Mean annual temperature ranges between 27-32°C. The soils of the zone have isohyperthermic temperature regime and receive average annual rainfalls of between 1600 mm-4338 mm [23].

### Observations and data collection

The soil samples used for the experiment were collected from 10 sites representing:

(a) Forests:

(i) An Artificial forest established by Forestry Department in 1962.

(ii) A Natural undisturbed forest (sacred land) that is more than 80 years old.

(iii) An Artificial *Gmelina arborea *forest established 30 years ago the Forestry Department.

(b) Grassland:

(i) Artificial grassland (golf course) established in 1934.

(c) Arable land

(i) Plot conventionally-tilled with traditional hoes, planted with cassava/vegetables/maize intercrop with 2-year fallow period in 10 years.

(ii) Plot conventionally-tilled with traditional hoes, continuously-cropped with maize/cassava/yam intercrop for ten years.

(iii) Plot conventionally-tilled, unmulched, cropped to maize and cassava for 12 consecutive years.

(iv) Land adjacent to the artificial forest cropped continuously for 15 years with cassava, yam, pulses and vegetables in a mixed culture.

(v) University Research plot, fallowed for two years and managed under conservation tillage for three years.

(vi) Farmers plot, conventionally-tilled, planted with cassava/maize/vegetables and used for 12 years.

The details of site number, location, soil classification and land use history are presented in Table 3.

An initial (reconnaissance) survey was carried out in the 10 sites selected for the study to establish sampling points. Nine representative sampling points were chosen in each selected site using the free survey approach (observation points that are representative of the site are chosen by the surveyors based on personal judgment and experience) [24]. Three sampling depths (0-5, 5.1-15 and 15.1- 30 cm) were used for the study. At each depth, nine undisturbed core samples and nine auger samples were collected for laboratory analysis.

The samples were collected at the end of the harvesting season in October when bulk density of tilled cropped fields had reverted to their pre-tillage conditions (because soil bulk density measurements are used for calculating carbon stocks) [17]. In cultivated plots, samples were collected randomly inside the rows. Auger samples were collected using a hand-pushed auger (Push Probe, 23 mm diameter). Core samples were collected using open-faced coring tube (area, 19.5 cm^3 ^and height, 5 cm from Eijkelkamp Agrisearch Equipment) at the three selected depths. Roots, twigs, and leaves were manually removed from auger samples and the samples air-dried at ambient temperature for 72 hours and subsequently sieved (using 2 mm sieves). Core samples were analyzed and mean results from each depth used whereas auger samples collected at a specific depth, were mixed and composite sub-samples (from each depth) used for further analyses.

The carbon stock in each agro ecological system was calculated with the formula = C (%)/100 × soil bulk density × area (1 ha) × soil depth

### Laboratory methods

Samples were analyzed in the Research Laboratory of the Department of Soil Science, University of Nigeria, Nsukka, for bulk density, gravimetric water content, organic carbon content, total nitrogen, soil pH and particle size distribution. Bulk density was analyzed by core method [25]. Organic carbon was determined by the Walkley-Black procedure [26]. Total nitrogen was by the Macro-Kjeldahl method [27], whereas soil pH on a saturated sample was determined in soil electrolyte (0.01 M CaCl_2_) suspension using a glass electrode pH meter (Digital pH meter, Accumet Model AR15, Fisher Scientific). Particle size distribution was determined using the pipette method of Gee and Orr [28].

## Competing interests

The authors declare that they have no competing interests.
